# Antisepsis in General Practice

**Published:** 1890-01

**Authors:** Thos. J. Charlton

**Affiliations:** Late house physician Bellevue Hospital, N. Y., Attending physician Savannah Hospital, of Savannah, Ga.


ARTICLE III.

### ANTISEPSIS IN GENERAL PRACTICE.

BY THOS. J. CHARLTON, M. D., LATE HOUSE PHYSICIAN BELLEVUE HOSPITAL, N. Y., ATTENDING PHYSICIAN SAVANNAH HOSPITAL, OF SAVANNAH, GA.

Although in the last few years many articles have been written on the subject of Antisepsis, and the remarkable results in surgery which follow a strict application of its methods, and although its merits are well recognized and applied by a majority of the profession in our larger cities and towns, still there are many among the profession at large, who do not give to this subject the attention that it merits. This, I think, can be justly attributed to two chief causes :

1. Most of the articles on this subject have been written by men connected with our larger hospitals, having every appliance and a corps of trained assistants at ready command. As a result of this, their descriptions of the necessary appliances and assistants have been elaborate and beyond the command of the average practitioner.

2. Many in applying the method neglect that attention to detail which is so necessary for success, and in failing to attain the results claimed, reject the method as being both unreliable and cumbersome.

My object in writing this article is not to present new meterial for consideration, but rather from the material at hand to endeavor to map out a simple and practical method of work by means of which perfect results may be obtained.

Antisepsis as denoted by the derivation of the word is that which acts against Putrefaction : Putrefaction, or Sepsis, being now considered as a form of fermentation produced in a tissue or fluid by the presence of micro-organisms and their resulting products. Septic infection of a wound is accidental and due to infection from without the body. When this does not occur or is prevented by the use of antiseptics, wounds heal without inflammation and there is no suppuration. Of all drugs possessing antiseptic properties Bichloride of Mercury and carbolic acid are by far the best for general use. These two possess the advantage of being thoroughly reliable, convenient to handle and obtainable everywhere. Just here I would caution against the use of the so-called antiseptic powders and solutions now offered for sale. These compounds are often attractive in appearance and agreeable in odor, but their antiseptic power is as an unknown quantity, and hence failure from their use is to be expected. It is far better to confine oneself to one or two antiseptics of known power, becoming familiar with their advantages and peculiarities, than to have an indifferent knowledge of many. The application of antiseptics to general practice is very simple, the appliances necessary are few and obtainable everywhere. The material used in the antiseptic treatment is as follows:

1. Concentrated solutions of Bichloride of Mercury and Carbolic Acid from the ordinary diluted solutions are made. The following is the formula of the Bichloride solution:

| | | |
|---|---|---|
| R | Hydrag. Bichloridi<br>Ammon. Muriat | } aa grs. 240 |
| | Aquae | Oj |

M. Sig. one-half ounce to a pint of water makes a solution 1-1000. The muriat of ammonia is added to this solution because it is thought to render it more stable and because, (as proved by Lister), in the presence of blood serum it increases its antiseptic power. Compressed tablets of this formula can now be bought at almost any drug store, and are more convenient to handle. The carbolic acid should be the pure crystalized and, before using, the bottle should be placed in hot water to liquify it. Six drachms of this acid to a pint of water makes a solution of 1-20, approximately. In addition to these solutions there should be an ordinary pepper box filled with iodoform.

Sutures and Ligatures. The materials most used are silk and catgut. Silk to be made antiseptic should be boiled in water for an hour and then kept in a well stopped bottle containing an alcoholic solution of bichloride 1-1000. Catgut is prepared by soaking it over night in bichloride Solution 1-1000, then in oil of juniper berries for twenty-four hours; after which it is transferred to absolute alcohol (95 per cent. alcohol answers well.) Catgut thus prepared is very strong and seldom breaks. In addition to silk and catgut, silver and horse hair are frequently used. For silver, all that is necessary is to heat it in the flame of a lamp until red hot and then transfer to carbolic solution 1-20 for a few minutes. For horse hair, select the coarse hairs from the tail of a horse; wash in chloroform or ether for a few minutes to remove all oily substances; then in water; keep in carbolic solution 1-20, and do not use until three or four days old. Horse hair makes a good drain for small wounds and is an excellent suture—its advantage being that a knot tied with it does not slip. Of the materials mentioned, silk is most generally used, and is now being used by many to the exclusion of everything else. It is not irritating and can be left in the tissue, as it rapidly becomes encysted and does no harm.

3. Drainage Tubing. The best material for general use is red rubber tubing, (this being of better quality than either the black or white). The tubing should be sufficiently stiff not to collapse from ordinary pressure. Most instrument makers have what they call defective catheters made of this material which they sell for about ten cents apiece. These make the best of drainage tubes and can be obtained in all sizes. Side openings should be made in the tubing about half an inch apart and large enough to permit of free drainage. It should be kept in 1.20 carbolic solution, which should be renewed from time to time.

4. Sponges. A very good sponge, answering all purposes, can be bought for about two and a half cents apiece. They should be carefully cleaned and kept in 1 20 carbolic solution. To bleach sponges, first remove all particles of sand; wash carefully in water; wring out the water, and place in a 1 per cent. solution of permanganate of potash for an hour—moving them about in the solution from time to time. Then, wring out this solution and place in a solution of hyposulphite of sodium (1 oz. to the gallon), to which 1 oz. of hydrochloric acid should be added. Keep them in this solution for about fifteen minutes, then remove them and wash carefully in water. An excellent substitute for sponges is absorbent cotton. Pieces of this, of convenient size, should be soaked in bichloride solution 1-1000 for a few minutes before being used.

5. Absorbent Dressing. For the dressing, ordinary cheese cloth, costing three to five cents a yard, answers well. The gauze should be boiled in a strong solution of caustic potash or soda to remove all oily substances. It should then be washed carefully in water to remove all trace of the alkali. Having done this, allow it to dry and then cut up into bandages. (If desired, the cloth may be bleached with chloride of lime.) Cut part of the cloth into five yard lengths, each piece being the full width of the cloth. Fold these in the direction of their width until reduced to a fold four to five inches wide and of the full length of the piece. Roll this up like an ordinary bandage. This is called a roller compress and is far more convenient to handle than a number of small compresses. Cut bandages of the ordinary sizes and roll as usual. Allow the compresses and bandages to soak for twenty-four hours in a Bichloride solution 1-1000; then, remove from the solution; cover with a clean towel wrung out in the same solution, and put aside to dry. When dry, keep in a well stopped glass fruit jar which has been previously washed out with the bichloride solution, 1-1000. Before using, the bandages and compresses should be again soaked in this solution for a few minutes. The reason of this is that gauze, like a sponge, is more absorbent when moist than when dry. The gauze may be treated with the bichloride solution before the bandages are cut. I do not think this is best, however, for after the gauze has been in the bichloride solution it should be handled as little as possible. Carbolized gauze may be made by soaking the bichloride gauze in carbolic solution 1-20 for a few minutes. Iodoform gauze may be made by sprinkling the bichloride gauze thickly with iodoform and rubbing it well into the meshes of the cloth. Iodoform, while generally used in antiseptic operations, has little or no antiseptic properties. Its action is due to its tendency to stop oozing and thus keep the wound dry.

6. Absorbent Cotton. This is usually applied over the bichloride dressing. Its antiseptic power is mechanical. It acts as filter, excluding from the dressing all particles of dust floating in the atmosphere. All the above preparations can now be obtained ready made from most drug stores. I would, however, advise that too much confidence be not placed in them when thus obtained, for made in large quantities by workmen ignorant of their application, carlessness is liable to occur in their manufacture and prove fatal to a good result. It is always best to make one's own dressing. It is but little trouble and the success which follows will amply repay it all. A good supply of all these preparations should be kept in a convenient bag, which should always contain a razor, a nail brush, safety pins, a few ordinary bandages, a tin pint cup and measure glass for the solution and a Davidson syringe for use as irrigator. Two ordinary tin bake pans should be strapped to the bag. These are for the instruments, needles, etc., which must be kept in carbolic solution 1-20 during the operation. Besides this, three or four Chinaware basins are necessary for the antiseptic solution used during the operation. These need not, however, be carried, as they can always be obtained at the home of the patient. All instruments used should be kept well polished and free from rust or dirt. They should have either metal or hard rubber handles baked on so as not to be affected by boiling water. All instruments, needles and safety pins for keeping the drainage tube in place should be boiled in water for at least half an hour and then placed in carbolic solution 1-20 for a few minutes before every operation of any importance. For the minor operations soaking in carbolic solution 1-20 for a few minutes will answer, provided they have been carefully cleaned before hand. The mode of procedure for an antiseptic operation is as follows: The physician and his assistants must carefully wash their hands and arms with soap and warm water, using a nail brush freely to remove all dirt from under the nails. After this, they are to be washed off with bichloride solution 1-500, a basin of this solution being kept convenient and used from time to time during the operation to rinse off the hands and arms. The part to be operated on should be carefully washed like the hands and if there is any hair on the parts it should be well shaved. The site of the operation is now surrounded with towels wrung out in bichloride solution 1-500. While the operation is going on the wound should be constantly irrigated with a warm bichloride solution 1-1000. Be careful that instruments, sponges, needles and dressings do not come in contact with the bed clothes or any other substance not disinfected. In case this should occur the instrument or dressing should be put aside and not used again during the operation. A Davidson syringe answers admirably for an irrigator, but in case it is not convenient, a sponge or piece of absorbent cotton answers well. It should, however, be soaked in bichloride solution 1-500 for a few minutes before being used with the irrigating fluid. As soon as the operation is completed all bleeding vessels should be tied and pressure made upon the wound with sponges wrung out in hot water, until all oozing ceases—as a dry wound favors an antiseptic result. As soon as all bleeding has ceased, the wound should be carefully washed out with irrigating fluid, dried with sponges and then dusted with iodoform. The drainage tube is now introduced and the wound stitched up. Introduce the stitches at close intervals and bring the edges of the wound into perfect apposition. Should there be much tension on the stitches a second and deep row should be introduced. These need not, however, be so near together. Replace the bichloride towels with fresh ones; fasten the drainage tube in place with safety pins, introduce as near the point of entrance and exit of the tube as possible; cut off the drainage tube close up to the safety pins; dust the surface of the wound with iodoform; place a piece of iodoform gauze around the ends of the drainage tube and between the safety pins and the wound, and cover these and the edges of the wound with a layer of iodoform gauze. Now apply two or more layers of the roller compress over the wound, extending it well beyond its edges. Hold this in place with a bichloride bandage over which an ordinary bandage should be placed. If necessary to keep the parts quiet apply a splint. If the patient is comfortable and free from pain and fever this dressing need not be disturbed for from two to four days, (depending upon the size of the wound); at the end of which time it is taken down to remove the drainage tube, after which the dressing is reapplied with the same precautions as at first. In removing a dressing it should first be thoroughly moistened with the irrigating fluid, and before the new dressing is applied, the wound should be carefully washed off with the same solution. In favorable cases the dressing is not again disturbed until union has taken place. In case oozing takes place through the dressing it should be taken down at once and reapplied. Should the wound become painful and the temperature rise the dressing should be removed; and if suppuration has occurred the tube should be left in or if removed it should be replaced and the wound washed out with bichloride solution 1-1000. This should be continued daily as long as there is suppuration and fever. When this ends, the drainage tube may be removed and a final dressing applied. A fresh wound treated thus will always heal by primary union unless the physician has been careless in his method or the patient affected with some constitutional trouble, as tuberculosis.

If called to a case in the country, and antiseptic dressings are not to be had, excellent results may be obtained by boiling the instruments, needles, ligatures and bandages in water for an hour. Wash the parts carefully with soap and water and use boiled water as an irrigating fluid. For drainage tube use a new lamp wick which has been boiled with the instruments, etc. In some of the hospitals in Germany, excellent results have thus been obtained. It is best, however, to replace this dressing with a more thoroughly antiseptic one as early as possible. No matter how dirty a wound may be when first seen it is possible to clean it and get an antiseptic result. The physician should always do his best. If the parts are much lacerated and contused do not stitch the wound up, but pack it loosely with iodoform gauze. Apply the dressing as described for wounds in general and be governed as regards the subsequent dressings by the condition of the patient.

In the treatment of an abscess cleanse the parts carefully with soap and water and wash off with bichloride solution 1-500, open the abscess and wash out its cavity with bichloride solution 1 1000. If the cavity is small, pack it loosely with iodoform gause and apply the antiseptic dressing. If the abscess is a large one, make an opening in its most dependent part; introduce a large size drainage tube; wash out the cavity and apply the dressing. The dressing should be applied daily until all suppuration ceases, when the tube may be removed and the final dressing applied. Ulcers can be successfully treated with this method. Wash the parts carefully and apply to the ulcer a solution of chloride of zinc—forty grains to the ounce—wash this off with bichloride solution 1-1000, and apply the antiseptic dressing. Repeat this daily until all suppuration ceases, then apply a final dressing.

Most brilliant results are obtained with this method in the treatment of compound fractures and in wounds opening into the joint cavities. With the old methods these injuries were usually considered as indicating either amputation, excision or resection. To day, with the antiseptic method, it is the rule for these cases to recover with useful limbs and moveable joints. In compound fractures the utmost care must be taken in cleaning the wound. If the bone cannot be replaced, saw off the projecting ends, reduce the fracture and wire the ends together. If the parts are much lacerated and contused do not sew up the wound but put in a drainage tube, (if necessary); pack the wound loosely with iodoform gauze, and apply the antiseptic dressing. This done, the wound should be put up in a plaster splint, an opening being cut out over the wound to facilitate dressing it. The dressing should be renewed as indicated by a rise of temperature, pain, or oozing of the discharges through the dressing. In injuries involving a joint cavity the wound should be carefully cleaned and, if necessary, enlarged. A drainage tube should be introduced and the joint washed out with either bichloride solution 1-2000, carbolic acid 1-20, or Thiersch's solution consisting of salicylic acid two parts, boric acid twelve parts, hot water one thousand parts. The antiseptic dressing is now applied and a splint put on. At the end of the third week passive motion of the joints should be commenced. If suppuration has taken place in the joint before it comes under treatment, an attempt should be made to arrest it. This is best done by first washing out the cavity with a solution of chloride of zinc—forty grains to the ounce; and then with bichloride solution 1-1000. This should be repeated daily until the suppuration disappears or until convinced that this procedure is of no avail.

In obstetrical practice the results which have followed the introduction of antiseptics are most brilliant. Puerperal fever, under its use, has almost entirely disappeared—its mortality being reduced at the present day to less than one-fourth of one per cent. In the New York Maternity Hospital the mortality for a period of nine years preceding the introduction of antisepsis was 4.17 per cent. of all women delivered. The mortality in the same institution for a period of five years following its introduction was 1.06 per cent. including death from all causes; the per cent. from sepsis alone was .27. During the years 1885 and 1888 not a death occurred in this institution from sepsis. This excellent result is due to the thorough antiseptic treatment introduced by Garrigues and most carefully carried out by his assistants. Corresponding results have been obtained in the maternity hospitals of our larger cities and in those of Europe using this method. While in private practice puerperal infection does not occur as frequently as in hospital practice, still the results obtained by the antiseptic treatment are such as not only to suggest its use in these cases but to demand it. Puerperal fever is to-day considered to be due to septic infection of the lochia by micro-organisms and to the absorption of this infection and its products into the general circulation through the channels laid open by the laceration which occurs, to a greater or lesser extent, in the genital tract of every woman confined. The chief source of infection is from particles of septic material adhering to the hands and instruments of the physician or nurse. Infection also takes place from septic dust floating in the atmosphere, but this is not so common.

The use of antisepsis in obstetrics is quite simple. The same attention should be given to cleaning and disinfecting the hands and arms as for antiseptic work in general. When labor begins the vagina should be carefully washed out with bichloride solution 1-2000, and during labor a basin of this fluid should be kept convenient for the attending physician to rinse his hands in before and after each vaginal examination. In making a vaginal examination it is not necessary to use oil or vaseline. Simply rinsing the hands in the bichloride solution is all that is necessary. After the child has been delivered the hand should never be introduced into the vagina unless some complication should arise requiring it. If the placenta is slow in coming away do not introduce the hand into the vagina to withdraw it, but with the hand on the abdomen grasp the fundus of the uterus, back of hand to rear, and knead it briskly, at the same time prizing it downward toward the hollow of the sacrum. When this is done prompt separation of the placenta takes place and it is quickly thrown off. After the placenta has come away, the vagina should be again washed out with the bichloride solution 1-2000. This injection should be hot (105 to 110 degrees), as it stimulates uterine contraction and thus tends to prevent post partum hemorrhage. For giving douches, the ordinary Davidson syringe is most convenient. To prevent the entrance of air into the vagina the suction end of the syringe should be kept well covered with the solution and all air should be expelled from the tubes and bulb by filling them with the solution. The syringe can be easily kept disinfected by the following method: When first called to a patient in labor place the syringe in a bichloride solution 1-1000, see that it is well covered with the solution and that its tubes and bulb is filled with it. After the syringe has been used wash it carefully in the bichloride solution and hang it up to dry before returning it to its case. After the douche following labor the external parts should be carefully washed off with bichloride solution and an occlusion dressing applied. This dressing should consist of two or more layers of the roller compress with an outer layer of absorbent cotton. The dressing should be large enough to cover the parts well and should be held in place by a piece of bandage fastened above and below to the binder. It should be renewed every three to six hours, (depending on the amount of the discharge), and should be kept up for ten days, or until the patient is up and about. In normal cases the vaginal douche need not be used, except as described above. While authorities differ in regard to the use of the vaginal douche before and after normal labor, the majority are in favor of the above plan. When the antisepsis is thorough the patient is free from fever, the lochia from fetor, and there is but little pain or soreness. Involution also seems to take place more promptly. Should the lochia become offensive and accompanied by an elevation of temperature, the vaginal douche should be used every three to six hours until the fever is reduced and the fetor disappears. If, notwithstanding the free use of the douche, the lochia continues offensive it is a sign that the uterus is infected, and the intra uterine douche is indicated. Except in cases where the placenta or membranes are retained and decomposing; in cases of still birth with maceration of the fœtus and offensive liquor amnii, and in cases in which it is necessary to introduce the hand or instruments into the uterus—the intra uterine douche should never be used. In using this douche great care must be taken and it should never be intrusted to a nurse. The best nozzle to use is of glass and can be very readily made from a piece of glass tubing twelve inches long and of a size larger than an ordinary lead pencil. Heat this in the flame of an alcohol lamp and bend one end of it until it approaches the curve of an ordinary urethral sound. Next, heat both ends of the tube to round off the rough edges, and put it aside to cool. Some recommend that side openings be made in the tube, but this is not necessary. When used this tube should be attached to a Davidson syringe and the same precautions taken as regards disinfection and exclusion of air as recommended for the vaginal douche. In using this douche the patient should be placed on her back with her hips drawn down to the edge of the bed—suitable precautions being taken to catch the water as it flows out and to prevent it from soiling the patient and the bed-clothes. With the patient on the back the danger of the injected fluid entering the cavity of the peritoneum through a dilated tube is lessened. To introduce the tube into the uterus pass two fingers ot the left hand into the vagina until they rest against the cervix. Now remove the hand from the vagina and place it on the abdomen over the fundus ot the uterus, keeping the tube in the median line, pass it gently forward until its end is felt near the fundus. Inject the fluid slowly and without force, and to enable it to flow out freely press the tube slightly upwards against the anterior wall of the cervex. The fluid used should be bichloride solution 1 4000. It should be hot (about 110 degrees) and should not exceed a quart. Before each intra-uterine douche a vaginal douche should be invariably given, and afterwards a suppository containing fifteen grains of iodoform should be introduced into the uterus. When it is suspected that the uterus contains particles of decomposing placenta or membranes an examination should be made, and if found present they should be removed with the finger or with a large blunt curette. It is much preferable to use the finger as there is less danger of injuring the uterine walls. When the above method is used early in the case the temperature promptly falls and with it the unfavorable symptoms are diminished and finally disappear. The douche should be given from every three to twelve hours, depending upon its effect in reducing the fever and in keeping it down. If the infection has become general before the case is seen the outlook is not so favorable, though when combined with the appropriate general treatment this method offers the best results.—*Southern Medical Record.*

### Editorial, Etc.

